# Design of a Tunable Snapshot Multispectral Imaging System through Ray Tracing Simulation

**DOI:** 10.3390/jimaging5010009

**Published:** 2019-01-05

**Authors:** Mengjia Ding, Peter WT Yuen, Jonathan Piper, Peter Godfree, Ayan Chatterjee, Usman Zahidi, Senthurran Selvagumar, David James, Mark Richardson

**Affiliations:** 1 Centre for Electronic Warfare, Information & Cyber, Cranfield Defence and Security, Cranfield University, Bedford SN6 8LA, UK; 2 Defence Science and Technology Laboratory (DSTL), Space and Sensing Systems Group, Porton Down, Salisbury, Wiltshire SP4 0JQ, UK

**Keywords:** coded aperture snapshot spectral imager (CASSI), snapshot imaging, optical system design, multispectral imaging

## Abstract

Research on snapshot multispectral imaging has been popular in the remote sensing community due to the high demands of video-rate remote sensing system for various applications. Existing snapshot multispectral imaging techniques are mainly of a fixed wavelength type, which limits their practical usefulness. This paper describes a tunable multispectral snapshot system by using a dual prism assembly as the dispersion element of the coded aperture snapshot spectral imagers (CASSI). Spectral tuning is achieved by adjusting the air gap displacement of the dual prism assembly. Typical spectral shifts of about 1 nm at 400 nm and 12 nm at 700 nm wavelength have been achieved in the present design when the air-gap of the dual prism is changed from 4.24 mm to 5.04 mm. The paper outlines the optical designs, the performance, and the pros and cons of the dual-prism CASSI (DP-CASSI) system. The performance of the system is illustrated by TracePro^TM^ ray tracing, to allow researchers in the field to repeat or to validate the results presented in this paper.

## 1. Introduction

Snapshot spectral imaging (SSI) through multiplexing of spatial-spectral information under compressive sensing (CS) principles has been a revolutionary advancement in remote sensing research [1,2,3,4]. It is achieved through the sparse property of natural scenery that exhibits strong correlations across the spectral and spatial domains. In CS, measurements of a signal are inner products between the signal and a set of vectors describing the measurement process, which can collectively be described as the measurement matrix. The measurement matrix is designed to have a particular property (the restricted isometry property) with respect to a matrix that transforms the signal into a basis in which its description is sparse. This makes it possible to retrieve the signal by solving a linear inverse problem, using many fewer measurements than would be required to guarantee perfect recovery of the signal according to the Nyquist theorem.

SSI not only overcomes the poor light collection efficiency of conventional slit-based hyperspectral imaging (HSI) [5], it is also capable of producing high quality spectral imagery with better signal-to-noise-ratio (SNR) [6] than that of the band filtering technique [7,8,9]. Over the last few decades of technological developments in the field, SSI technology has become mature enough for commercial exploitation [10,11]. There are a variety of approaches to realize SSI, such as the computed tomographic imaging spectrometer (CTIS) for astronomical application [12] using a diffracted multiplex projection through the computer-generated holographic disperser [13] or a 2D diffraction grating [14]; the image-replicating imaging spectrometer (IRIS) [15,16] which employed a cascaded birefringent two-beam interferometers to realize polarized dispersion; and the coded aperture snapshot spectral imager (CASSI) [17,18,19,20,21] and derivatives [22,23,24,25,26] which exploited compressive sensing [12] by deploying a spatial and a spectral modulator.

One limitation in most SSI systems reported to date is the fixed set of exploitable wavelengths [15,18,20,25,27,28,29,30,31] that can be recovered from the data for a given configuration of the optics in the system. Although spectrally tunable SSI systems have been achieved using multiple shots and varying code word designs [32], or doubly-encoded systems [31], this paper proposes an alternative approach to realize spectral tuning through electro-optical (EO) hardware. The spectral tunability in this work is realized by using a dual prism assembly as the dispersion element within CASSI architecture and the paper aims to illustrate the feasibility of wavelength tuning in SSI through a simple EO design.

## 2. Background of Coded Aperture Snapshot Spectral Imaging

The CASSI is a remarkable snapshot multispectral architecture that has been widely studied in the past decade [3,10,11]. Its very first version [17] was a Dual Disperser CASSI (DD-CASSI) structure that utilized two prisms, one to disperse and the other to “de-disperse” the light. Subsequently the single disperser CASSI (SD-CASSI) [18] was proposed. Various versions of the SD-CASSI designed with enhanced performances have been reported [19,20,25,30,33,34,35]. The SD-CASSI is an architecture that consists of one coded aperture mask for the spatial modulation and one diffraction element for the spectral dispersion. Under SD-CASSI, the 2D detector acquires a multiplex of spectral-spatial data and the multispectral image of the scene is recovered using compressive imaging principles [1,36,37]. Other variants of CASSI includes the 3rd generation SD-CASSI [33], which utilized multiple code words through a piezo-driven coded mask for enhancing reconstruction accuracy and spectral resolution [33]. The extension of spectral range into the ultraviolet [20], enhanced decompression algorithms [34], and video rate SSI [35] have been reported in the last few years.

**Figure 1:**
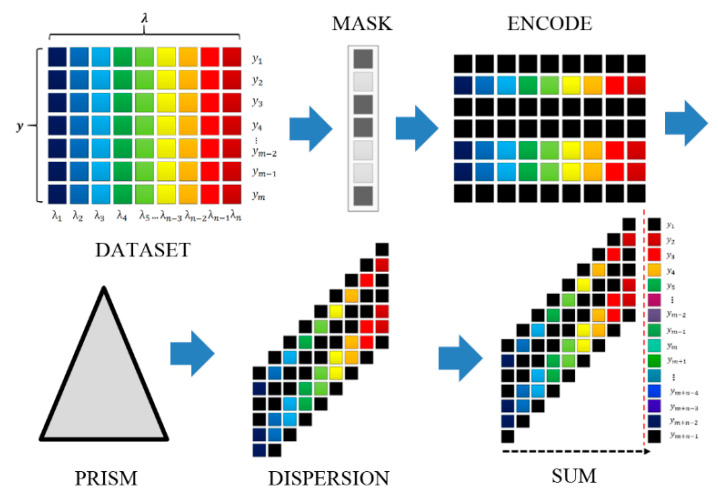
Schematic of Coded Aperture Snapshot Spectral Imaging (CASSI) showing three stages in the “Mask-Prism” model: The original multispectral data are encoded by an array of coded aperture mask, the encoded dataset is sheared by the prism, and eventually the spatial-spectral data is integrated at the detector in a multiplex manner.

The principle of spatial spectral multiplexing in SD-CASSI is schematically shown in Figure 1, which depicts a simple “Mask & Dispersive element” as the building block of the SSI system. The mask is a simple pinhole array with closed and open pinholes in a plane that spatially modulate the transmitted light reflected from the scene. The selectively transmitted pattern is then dispersed into individual spectral channels, which are then projected onto the pixels of the focal plane array detector. The properties of the mask pattern and dispersive element are important for balancing the tradeoff between spatial and spectral resolutions.

**Figure 2:**
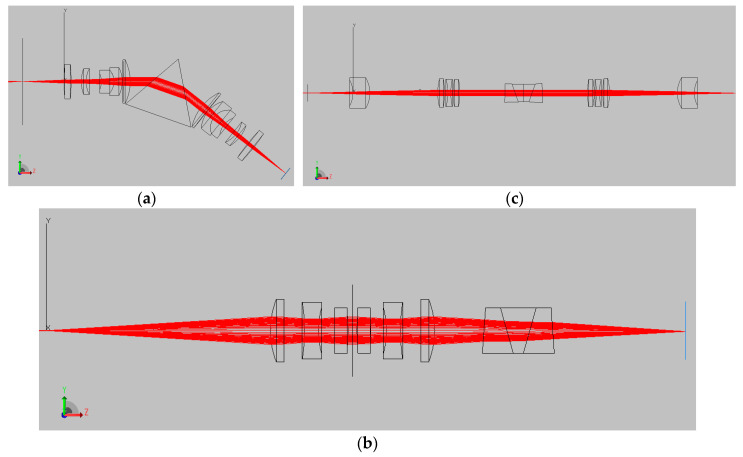
Example of ray propagations for three versions of Coded Aperture Snapshot Spectral Imager simulated by TracePro^TM^ (**a**) Single Disperser CASSI; (**b**) Double-Amici CASSI; (**c**) Ultra-Violet CASSI (see references [18,19,20]).

Common to all spectral imaging system, the dispersive element design is one of the most influential factors on the performance of SSI system. In the 1st generation of SD-CASSI, an equilateral prism was employed, which exhibited strong anamorphic distortions in both spectral and spatial domains due to the wavelength-dependent dispersion of the equilateral prism. Subsequently, a custom-designed double amici prism with off-the-shelf relay optics was utilized in the 2nd generation SD-CASSI, which showed an improved spectral resolution [19]. The evolution of the CASSI framework is shown in Figure 2 [18,19,20], developing from SD-CASSI based on the equilateral prism, Double-Amici CASSI with a direct view, and the latest Ultra-Violet CASSI serving a larger spectral range. This paper proposes a simple modification of the CASSI frame—using a dual-prism CASSI (DP-CASSI) as the dispersive element to study how the spectral properties of the system can be tuned by modulating the air gaps between the dual prisms.

## 3. Dual-Prism Coded Aperture Snapshot Spectral Imaging System

For a given coded aperture M(x,y) with square holes of side lengths $\Delta_{C}=\;q\Delta_{D}$, where $\Delta_{D}$ is the detector pixel pitch, and *q* is an integer showing the magnified relationship between coded aperture pixel pitch and detector pixel pitch, the spectral density of the scene $f_{0}\left(x,y,\lambda \right)$ modulated by *M* is given by:

(1) $$f_{1}\left(x,y,\lambda \right)=\;f_{0}\left(x,y,\lambda \right)M\left(x,y\right),$$

where (x,y) are coordinates in the spatial dimension and x, y, λ∈ℛ, 0≤x≤m, 0≤y≤n, 0≤λ≤l. The double prism disperses light along one dimension, say, in the *x*-direction, which induces the spectral content of the scene to shear along the *x*-axis. Given the effective dispersion coefficient of the double prism N(λ), the dispersion of the double prism D(λ) with respect to the center wavelength $\lambda_{c}$ in the spectral range is $D\left(\lambda \right)=N\left(\lambda \right)\left(\lambda -\lambda_{c}\right)$. The image of the detector g(x,y) results in a multiplex of modulated spectral and spatial contents along the x-axis, which can be written as:

(2) $$g\left(x,y\right)=\int^{\text{​}}f_{0}\left(x+D\left(\lambda \right),y;\lambda \right)T\left(x+D\left(\lambda \right),y\right)d\lambda .$$

The measured spectral density at the detector integrates the spatial shift of spectral channels λ{1…l} and the modulated spatial content of the scene over the spectral range of the system [3].

The spectral resolution is mainly constrained by the dispersion capability of the prism and the number of resolved spectral bands $l=p\left[\frac{\lambda_{1}-\lambda_{L}}{\Delta_{C}}\right]$, where [•] represents the maximum integer value and p represents sampling ratio at detector. Therefore, Equation (2) can be written as:

(3) $$g_{m,n+l-1}=\;\sum_{k=1}^{l}{\left(f_{k}\right)}_{m,n+k-1}T_{m,n+k-1}+\omega_{mn},$$

and in operator form, where *T* is replaced by the operator H:

(4) g=Hf+W,

where $H\in {ℛ}^{\left[m•\left(n+l-1\right)\right]\times \left(m•n•l\right)]}$ denotes the observation operator, $f\in {ℛ}^{\left[\left(m•n•l\right)\times 1\right]}$ and $g\in {ℛ}^{\left[m•\left(n+l-1\right)\right]\times 1)]}$ denote the detector measurement matrix and the recovered signal respectively, and W takes into account all possible noise sources [18]. $g_{mn}$ is the multiplex measurement version of $f_{mnk}$ in discrete form. If the spectral data cube $f_{mnk}$ can be further expressed as the continuous form f=ωξ where ω is, for example, the inverse wavelet transform and ξ is the three dimensional coefficient wavelet decomposition of **f**, then Equation (3) can then be rewritten as:

(5) g=Hωξ+W,

where the linear operator H represents the system forward model. The reconstruction of **f** is attained by solving the reconstruction algorithm to recover **f** from g through the optimization of the linear inverse problem (LIP):

(6) $$\hat{f}\left(\tau ,\Upsilon \right)=\underset{\xi }{\mathrm{argmin}}\left[\frac{1}{2}\|g-H\omega {\xi \|}_{2}^{2}+\tau \|\Upsilon {\left(\xi \right)\|}_{1}\right],$$

where g is the measurement data of the dual-prism system and system forward model H accounts for the effects of the coded aperture and the dispersion by the dual prism. The first term minimizes the $l_{2}$ difference between the model and the measurement g. The variable τ>0 is the regularization parameter that balances the conflicting tasks of minimizing the least square of the residuals and at the same time to yield a sparse solution. It can be seen that the sparser the source f in ξ, the better the performance of the reconstruction algorithm. Υ can be in various forms and the total variation (TV) regularization [38] is adopted here for better spatial smoothing. The TV regularizer has a discrete formation given by:

(7) $$\Upsilon_{\mathrm{TV}}\left(f\right)=\sum_{i}\sqrt{{\left(\Delta_{i}^{h}f\right)}^{2}+{\left(\Delta_{i}^{v}f\right)}^{2}},$$

where $\Delta_{i}^{h}$ and $\Delta_{i}^{v}$ denote discrete gradient operators in the horizontal and vertical direction, respectively. The regularization product is in the $l_{1}$ norm, which can be minimized by exploiting Chambolle projection’s algorithm [38] to compute the projection of weighted estimate from the convex data set. The weight of the tuning parameter τ and the solution $\hat{f}$ in Equation (6) affects the balance between spatial smoothness and the spectral recovery fidelity.

There are a number of strategies [39], such as the gradient projection for sparse reconstruction (GPSR) algorithm [40], SPGL1 [41], large-scale L1 (LSL1) regularized least squares [42] and two-step iterative shrinkage/thresholding (TwIST) [43] which have been proposed for solving the LIP in Equation (6). For this work, we adopted the TwIST algorithm due to its fast convergence rate. For a linear system, Ax=b TwIST firstly decomposes A such that A=C−R where C is positive definite and easy to invert, and a two-step procedure has been proposed for solving the LIP:

(8) $$x_{1}=x_{0}+\beta_{0}C^{-1}\left(b-Ax_{0}\right),$$

(9) $$x_{t+1}=\left(1-\alpha \right)x_{t-1}+\alpha x_{t}+\beta C^{-1}\left(b-Ax_{t}\right),$$

for t≥1, $x_{0}$ is the initial vector, where $\alpha ,\;\beta ,\;\beta_{0}\;$ are the parameters of the algorithm. Note that the “two-step” stems from the fact that the iteration depends on both $\;x_{t}\;$ and $\;x_{t-1}\;$ and it has been proved that TwIST achieves much higher convergence rate than the IST algorithm [43].

### 3.1. Dual-Prism and Relay Optics Design

**Figure 3:**
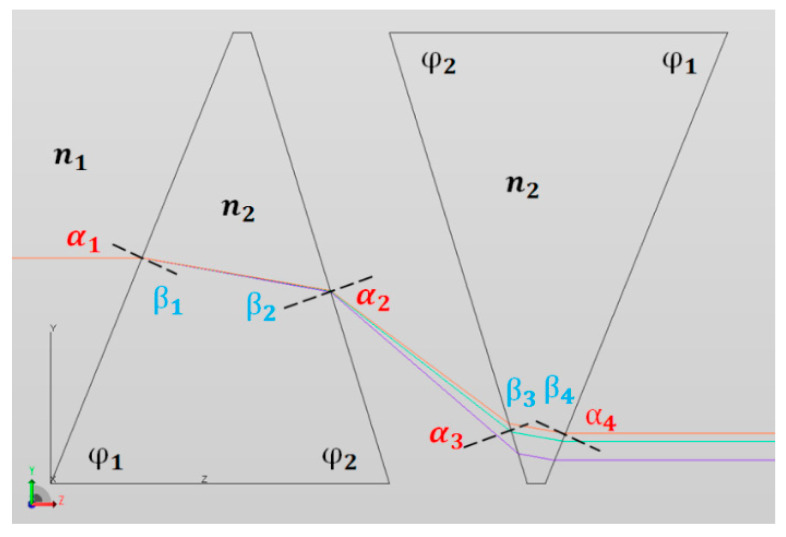
On-axis ray propagations of the dual-prism system for prism angles *φ*_1_ and *φ*_2_. Note that the on-axis exit ray is parallel with that of the incoming ray; and the 3 colored rays (purple, green, and red) represented the short (450 nm), mid (550 nm) and long (700 nm) wavelengths, respectively.

Dual-prisms have been widely utilized for electro-optics applications such as the introduction of group-delay dispersion to compensate the chirp of ultrashort laser pulses [44]. This work utilizes a pair of identical triangular prisms such that the directions of propagation of the rays are unchanged and a relative dispersive displacement in the sagittal plane is generated, as shown in Figure 3. The present system consists of a set of dual-prisms with fundamental angles of $\varphi_{1}=67.64{}^{\circ}$ and $\varphi_{2}=83.48{}^{\circ}$. The two prisms are separated by adjustable air gaps of 4–5 mm.

The ray optics of the prism assembly shown in Figure 2 accord with the following behavior:

(10) $$\mathrm{First}\;\mathrm{prism}:\;n_{1}sin\left(\alpha_{1}\right)=\;n_{2}sin\left(\beta_{1}\right),$$

(11) $$\beta_{1}+\beta_{2}=180{}^{\circ}-\left(\varphi_{1}+\varphi_{2}\right),$$

(12) $$n_{2}sin\left(\beta_{2}\right)=\;n_{3}sin\left(\alpha_{2}\right).$$

(13) $$\mathrm{Second}\;\mathrm{prism}:\;n_{1}sin\left(\alpha_{3}\right)=\;n_{2}sin\left(\beta_{3}\right),$$

(14) $$\beta_{3}+\beta_{4}=180{}^{\circ}-\left(\varphi_{1}+\varphi_{2}\right),$$

(15) $$n_{2}sin\left(\beta_{4}\right)=n_{1}sin\left(\alpha_{4}\right),$$

where the refractive indices of air and in the prism are $n_{1}$ and $n_{2}$, respectively.

**Figure 4:**
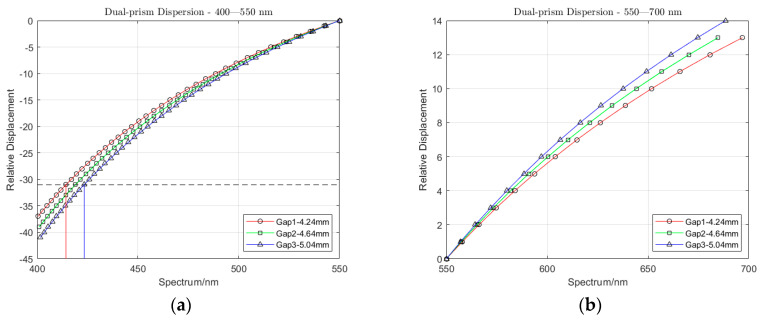
The dispersion of the dual-prism for three air gaps of 4.24 mm, 4.64 mm, and 5.04 mm in the spectral region of: (**a**) 400–550 nm, (**b**) 550–700 nm. The *y*-axis presents the location of pixels along the dispersion plane with respect to the pixel that senses the 550 nm wavelength (the central wavelength position) which is set at 0 mm.

Note that the two prisms are identical and parallel to each other, so $\alpha_{3}=\alpha_{2}$ similarly $\alpha_{1}=\alpha_{4}$. Additionally, the incident angle at the entrance is dependent on the prism angle *φ*_1_ only. The dispersion of the dual-prism at a specific air gap can be calculated through Equations (10)–(15). Given a dual-prism of type N-SF11 with $\varphi_{1}=67.64{}^{\circ}$ and $\varphi_{2}=83.48{}^{\circ}$ at air gaps of 4.24 mm, 4.64 mm and 5.04 mm, the displacements in the sagittal plane for the spectral range 400–700 nm relative to the center wavelength of 550 nm is shown in Figure 4. Note that the data (Figure 4) is shown for vertical displacement intervals of 6.5 μm, which was set to be the same as the pixel pitch of the sCMOS PCO sensor considered in this paper. In other words, the *y*-axis represents the pixel positions of the detector which senses the dispersed light in the sagittal meridional plane of the optics. Similar to all spectral sensing systems, the various spatial locations of pixels along this axis sense a specific wavelength of the dispersed light. The curvature shows that the dispersion is not linear in the spatial dimension, which is due to the simplistic optical design of the diffraction element employed. It should be emphasized that Figure 4a presents the wavelength-tuning characteristic of the DP-CASSI—the horizontal line indicates the spectral shift across the three curves when the air gap is changed from 4.24 mm to 5.04 mm. The spectral shift at 400 nm is about 1.1 nm and converges to zero shift at 550 nm, which is regarded as the origin reference point. In the red region (above 600 nm), the spectral shift due to the change of air gap is found to be much larger, varying between 2 and 12 nm. This is due to the fact that the shorter wavelength has larger dispersion than that of the long wavelength spectral region, resulting in more dispersed information in the shorter wavelength region.

### 3.2. System Design

**Figure 5:**
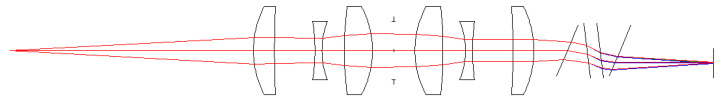
Ray diagram of the Dual-Prism Coded Aperture Snapshot Spectral Imager simulated by the OSLO lens design software.

The relay optics of the DP-CASSI was optimized by optical design software OSLO^TM^ to minimize the Seidel aberration. The first three elements utilize a Cooke triple that effectively produces apochromatism. The ray optics of the system were simulated by TracePro^TM^ (Lambda Research Corporation, MA, USA) using a bundle of incident rays of wavelengths 400–700 nm. The overall snapshot imaging system contains imaging optics, relay optics and dispersive elements. In this paper, we only simulated the performance of relay optics and dispersive elements. The ray diagram (Figure 5) illustrates the propagation of chromatic rays in the relay optics and dual-prism with the object plane on the left hand as the starting point and the coded aperture is placed at the object plane.

**Figure 6:**
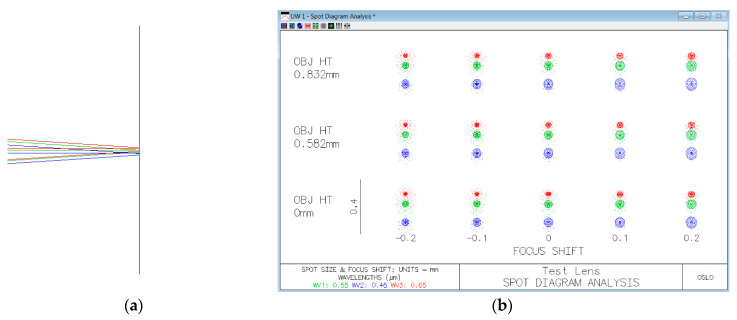
Focus condition of the dual-prism CASSI at the image plane: (**a**) ray diagram for the 450, 550, and 650 nm presented in blue, green, and red colored rays respectively; (**b**) the spot diagram of 450, 550, and 650 nm rays at three different field points and five focus shifts with respect to the focal point.

Figure 6a shows the zoom-in screenshot of the long, mid and short wavelength of rays between 400–700 nm in red, green, and blue colors after the dispersive element respectively. It is seen that the re-imaging rays are focused rather well at the sensor plane. The spot diagram presented in Figure 6b was evaluated by the OSLO software, and it shows the spatial distribution of the rays at 450, 550, and 650 nm, going through the entrance pupil and forming on-image plane at three object heights (on-axis, 0.582 mm and 0.832 mm). The spectral dispersion is clearly seen, which is of great importance to separate spectral information in the spatial domain and then apply estimation algorithms to recover the independent components from a multiplex image.

The dispersive optics assembly is conventionally placed in the collimating space, to alter collimated rays’ direction in order to acquire the corresponding dispersive displacement formed on the detector by the optics. When the dual-prism follows this structure, it did not result in any spectral dispersion after re-imaging onto the detector. This is due to the dispersion feature of the dual-prism that produces the wavelength-dependent lateral dispersion without altering the incident ray’s direction. In this work, the dual-prism was placed within the focal range of the relay optics to take advantage of the focusing (by the relay optics), and thus form a focused image. However, the addition of a dual-prism introduces aberrations, which were reduced by using a smaller numerical aperture and optimizing the optics to minimize the problem. The optimization of the optics was done using OSLO software.

## 4. DP-CASSI Spatial Spectral Multiplex Imaging Simulation

### 4.1. System Setup

**Figure 7:**
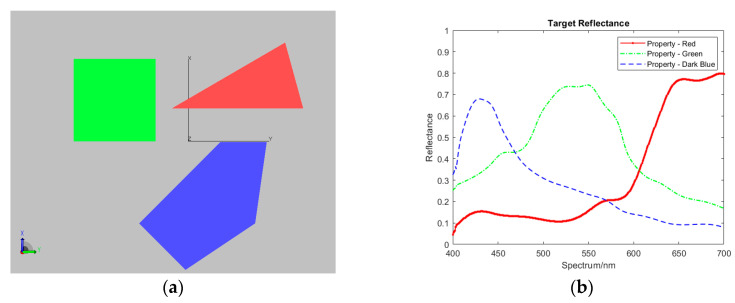
Samples of 3D targets for testing: (**a**) RGB presentation. (**b**) Spectral reflectance characteristics of the three targets.

**Figure 8:**
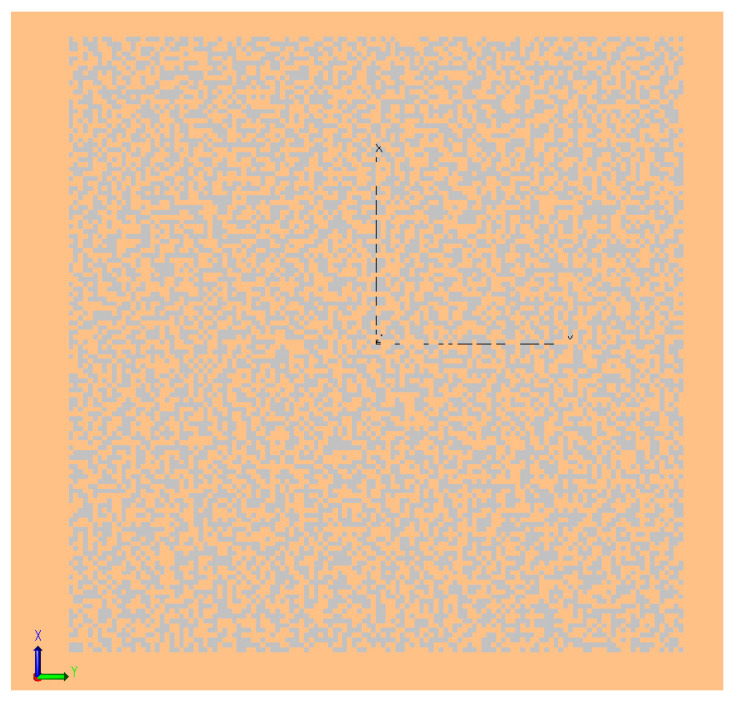
False color map of the random 128 × 128 coded aperture with the smallest aperture of 13 μm. The mask was constructed using the “perfect absorber” property with geometric sizes of 1.664 × 1.664 mm.

The imaging of scenes using the DP-CASSI was simulated by TracePro^TM^ under synthetic illumination sources. Three solid, colored objects in the shape of a square, triangle, and pentagon, with surface properties in the 400–700 nm spectral range were designed in Matlab, as depicted in Figure 7. The size of the targets was limited to the scale of the coded aperture, which was a 3 × 3 mm chrome-based metal mask with a central active pattern area of 1.664 × 1.664 mm. The code word was generated by randomly shuffling a core pattern of $\left[\begin{array}{cc} \begin{array}{cc} 0 & 1\\ 1 & 1 \end{array} & \begin{array}{cc} 0 & 1\\ 0 & 1 \end{array}\\ \begin{array}{cc} 0 & 1\\ 1 & 0 \end{array} & \begin{array}{cc} 0 & 0\\ 1 & 0 \end{array} \end{array}\right]$ 32 times, where zeros represent opaque areas and ones represent an open area, with a width of 13 μm for each element, giving a 50% randomly open pattern as depicted in Figure 8.

**Figure 9:**
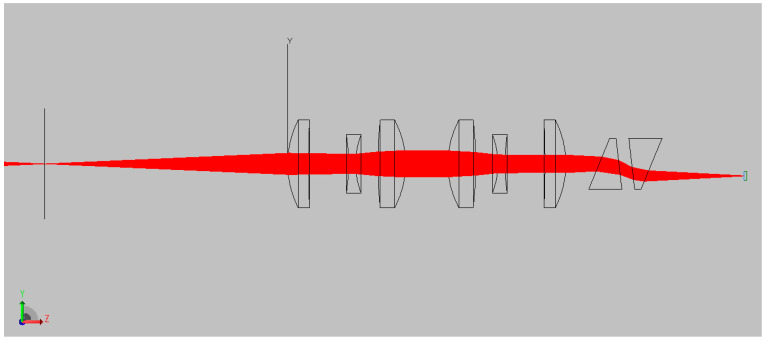
Ray diagram of the DP-CASSI showing how the 650 nm wavelength propagates through the coded aperture (far left), the six-element relay lens in the middle, the dual-prism, and the focal plane array (FPA, far right).

Figure 9 illustrates the ray propagation throughout the DP-CASSI, with a point source at 650 nm, modeled in TracePro^TM^. The illumination source used in the simulation consisted of 61 wavelengths uniformly spread between 400 and 700 nm. Due to the small numerical aperture, the light source directly emitted a collimated grid source of 65,000 rays per wavelength to the target, which bounce back towards the coded aperture. The detector (PCO SCMOS) which has dimensions of 3.328 × 3.328 mm with 512 × 512 mesa and a 6.5 μm pitch, was constructed as a perfect absorber of solid objects with a top-hat quantum efficiency of 100% across the spectral region of interest (ROI). This design ensured a field of view of 1.664 × 1.664 mm at the coded aperture, with a minimum resolution of 6.5 μm.

### 4.2. Spectral Calibration

**Figure 10:**
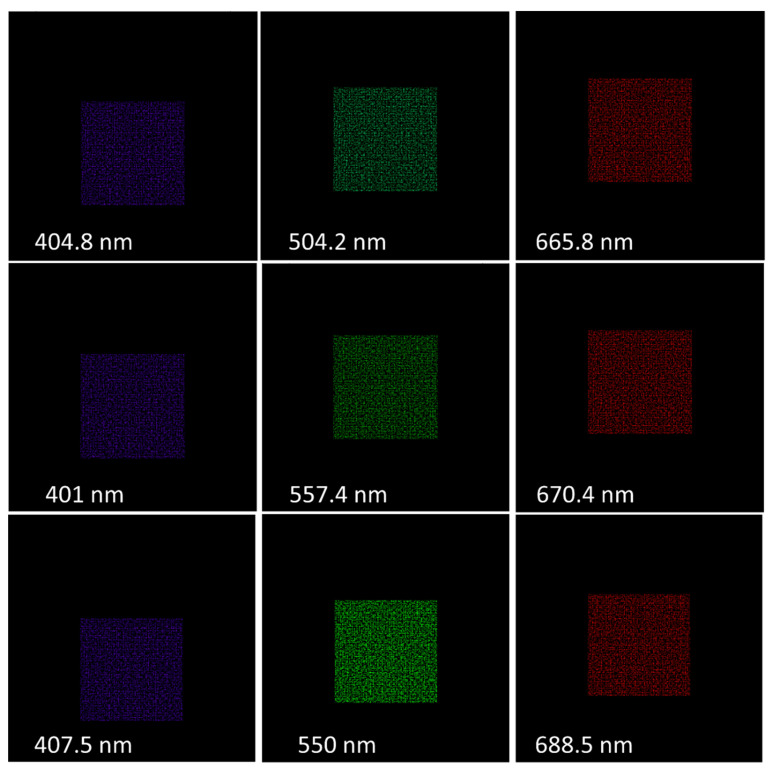
Images of the coded aperture mask using the TracePro^TM^ color scheme for a selection of six calibration frames under sequential illumination of monochromatic light for three air gap distances of the dual-prism: 4.24 mm (**upper row**), 4.64 mm (**middle row**) and 5.04 mm (**bottom row**).

**Figure 11:**
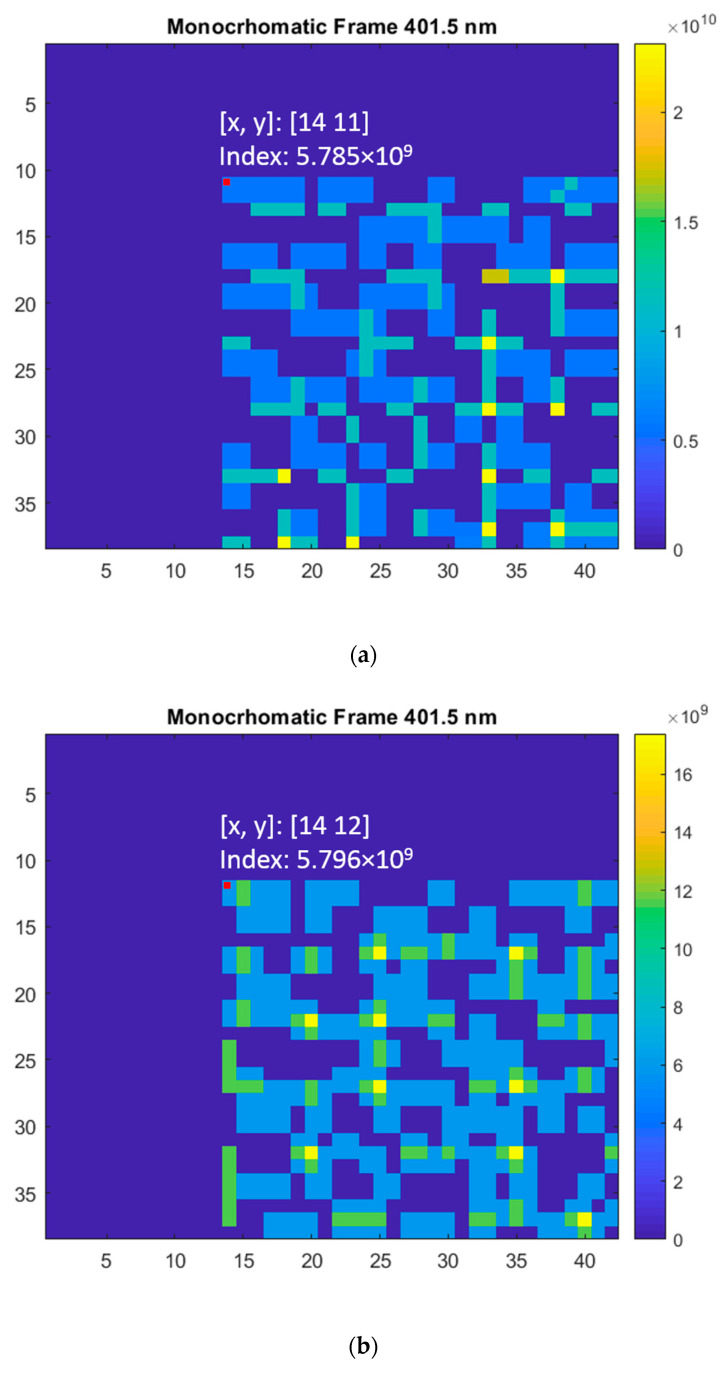

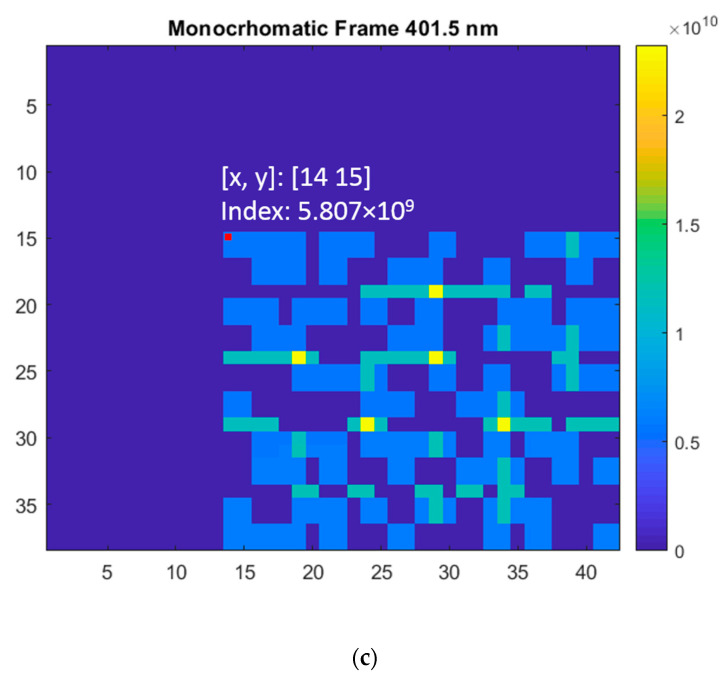
The false color intensity image of the coded aperture with illumination by monochromatic light of 401.5 nm wavelength at air gaps of (**a**) 4.24 mm, (**b**) 4.64 mm, and (**c**) 5.04 mm. The intensity maps of the three patterns are different due to the different light dispersion conditions at these three air gap displacements in the DP-CASSI system.

Like all spectral imaging devices [5], the DP-CASSI requires a spectral calibration procedure to identify the spatial-spectral characteristics of the system. Spectral calibration in DP-CASSI was performed using monochromatic light sources with calibrated wavelengths, as shown the example in Figure 10. Each of these wavelengths sequentially irradiated the coded aperture mask under the same conditions as that of the imaging experiment. The spatial positions of every pixel in this set of coded aperture images gave a characteristic spatial-spectral relationship like that shown in Figure 4, which were then used as a measurement matrix for multiplex data decompression. Figure 11a–c illustrate an example of the spatial-spectral relationships of the DP system illuminated by 401.5 nm monochromatic light at three different airgap displacements of 4.24, 4.64 and 5.04 mm respectively. The figure is presented using a false color intensity map, and they are seen to be slightly different due to the slightly different dispersion conditions of the light through the DP system at the three different airgap positions. For the 6.5 µm pitch of the FPA detector utilized in this work, simulation experiments showed that the same pattern (in the ~400 nm region) is obtained when the airgap displacement is smaller than 0.09 mm.

### 4.3. Simulation of Image Formation by TracePro^TM^

**Figure 12:**
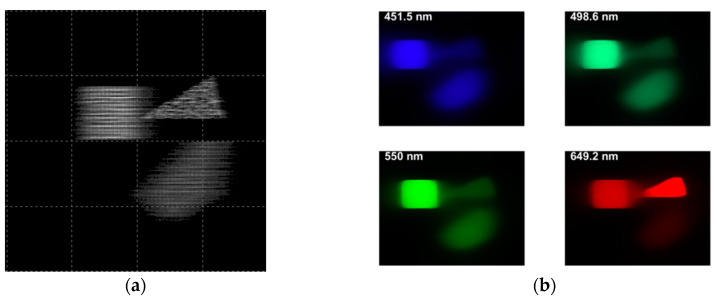
Sample results of the DP-CASSI system at an air gap of 4.24 mm: (**a**) spatial-spectral multiplexed raw grey scale image of the three targets (**b**) color images of the scene in CIE 1964 color scheme at four different wavelengths reconstructed through the linear inverse equation (Equation (6)) using regularizer *τ* = 0.5.

**Figure 13:**
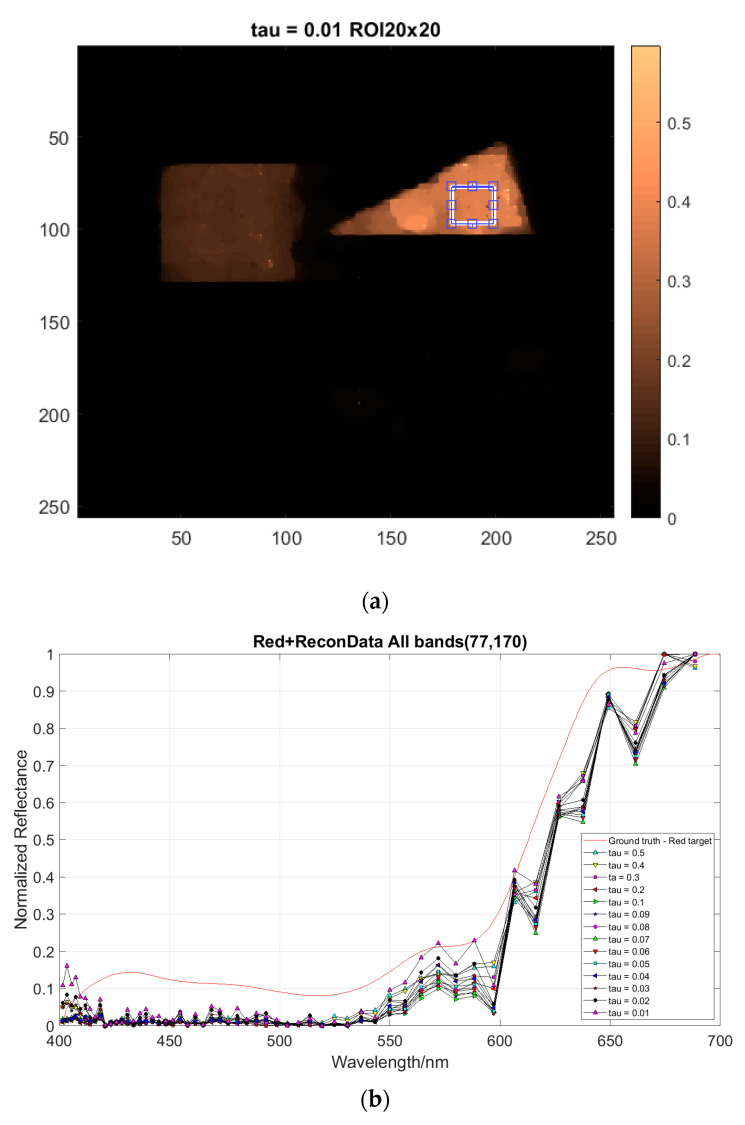
The effect of reconstruction for (**a**) the red triangle target using a range of (**b**) the regularizer parameter *τ* 0.01–0.5. The smaller the values of *τ* (e.g., 0.02 and 0.01) give better spectral reconstruction accuracy.

Simulation of imaging by the DP-CASSI system with three colored objects in the scene under three different air gaps of the dual prism (4.24, 4.64, and 5.04 mm) was carried out using TracePro^TM^ (see Section 4.1 and Section 4.2 above). The multiplexed image (Figure 12a), together with that decompressed using the TwIST algorithm were determined using the procedure as reported in the paper [45]. The model parameters were as follows: τ between 0.01 and 0.5 (Figure 13), number of iterations =50, TV iterations =4, and (α, β) from Equation (9).

The quality of the images shown in Figure 12b is seen to be fair and exhibits a certain degree of blurring, which can be attributed mainly to the uncorrected optical aberration of the optics in the system. The objective of the present work was to illustrate the effectiveness of wavelength tuning in the SSI system through the optical approach, and optical optimization such as aberration will be addressed in the next phase of the work.

### 4.4. Regularization

**Figure 14:**
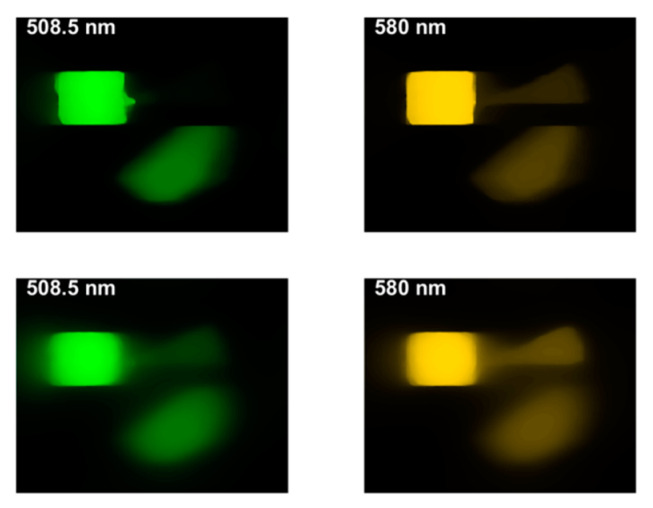
The effects of the tuning parameter τ in the TwIST algorithm for two wavelengths at 508.5 and 580 nm: (**upper panel**) *τ* = 0.1 and (**lower panel**) *τ* = 0.5. The aberration is stronger for larger *τ*.

The regularization parameter τ in Equation (6) balances the optimization between the spatial resolution and the spectral accuracy of the multiplex image decompression. It is seen that the larger value of τ (e.g., τ=0.5) gives more spectral errors (see Figure 13), while at the same time exhibiting a higher degree of spatial smoothness (see Figure 14). The correct regularization parameter could in principle be optimized under certain constraints; however, this will be developed in the next phase of the work. The regularizer τ=0.1 was used here to illustrate the wavelength tuning.

### 4.5. Spectral Tuning by Variation of the Air-Gap in the Dual-Prism

**Figure 15:**
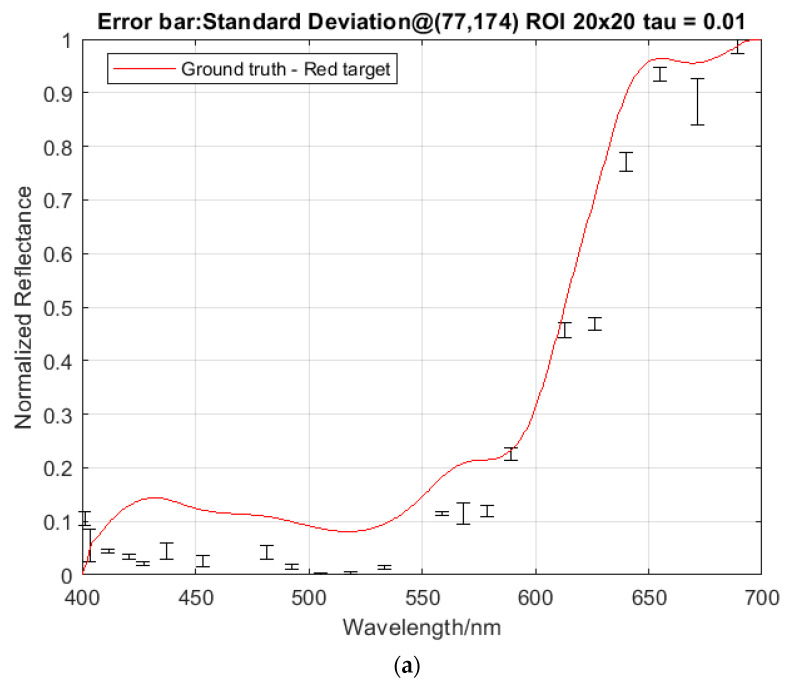

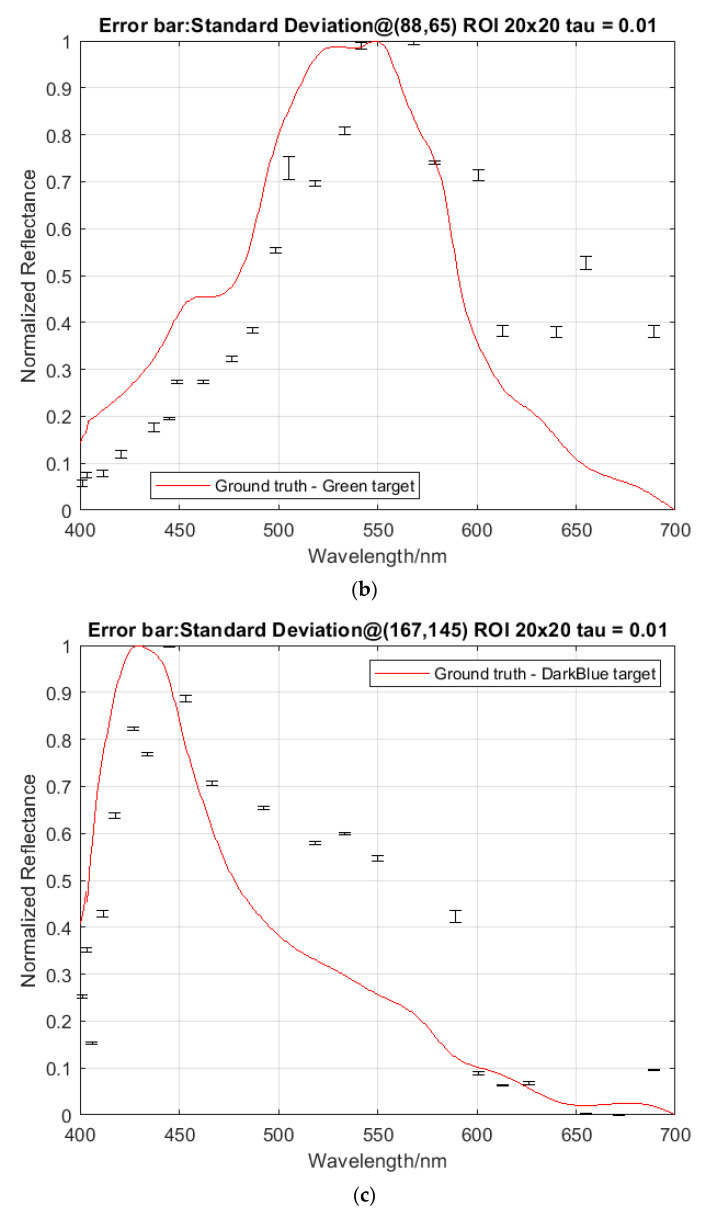
The accuracy of spectral reconstruction for the three targets using combined results of three different air gaps are shown. In all cases, the reconstruction data is the mean of a 20 × 20 pixel ROI with standard deviation as error bars and compared with that of the ground truth spectra (solid line): (**a**) red triangle, (**b**) green square, (**c**) blue pentagon.

**Figure 16:**
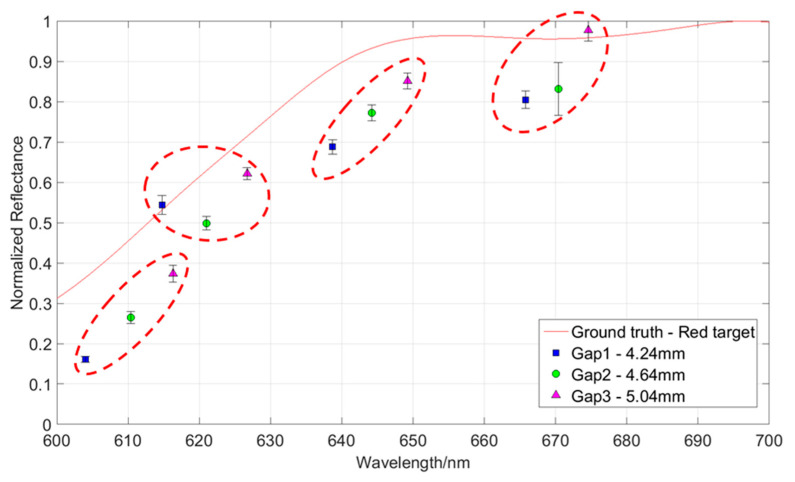
Four groups of reconstructed spectral characteristics for the red triangle showing the wavelength tuning of the DP-CASSI with respect to the corresponding air gaps.

The spectral tuning in DP-CASSI can be interrogated by tracking the spectral wavelengths of a specific pixel when the air gap displacement of the dual prism is modified. As depicted in Figure 4, the same pixel will sense a different wavelength of light when the air gap of the prism changes. Figure 15 plots a few normalized reflectance averaged over a 20 × 20 px region for one air gap simulation in three different colored data points. The error bars shown in the figure are the standard deviation over the 400 pixels in the selected ROI. Note that the recovered spectra of the object (red colored target) does not completely match with that of the ground truth data. This is believed to be partly due to the optical aberration and partly to the inefficiency of the decompression algorithm (TwIST). Figure 16 plots the reconstructed spectra in the region 600–700 nm of the red triangle for the three different air gap simulations, together with the ground truth data for direct comparison. The wavelength shifts across three sets of data is tabulated in Table 1.

## 5. Discussion

**Figure 17:**
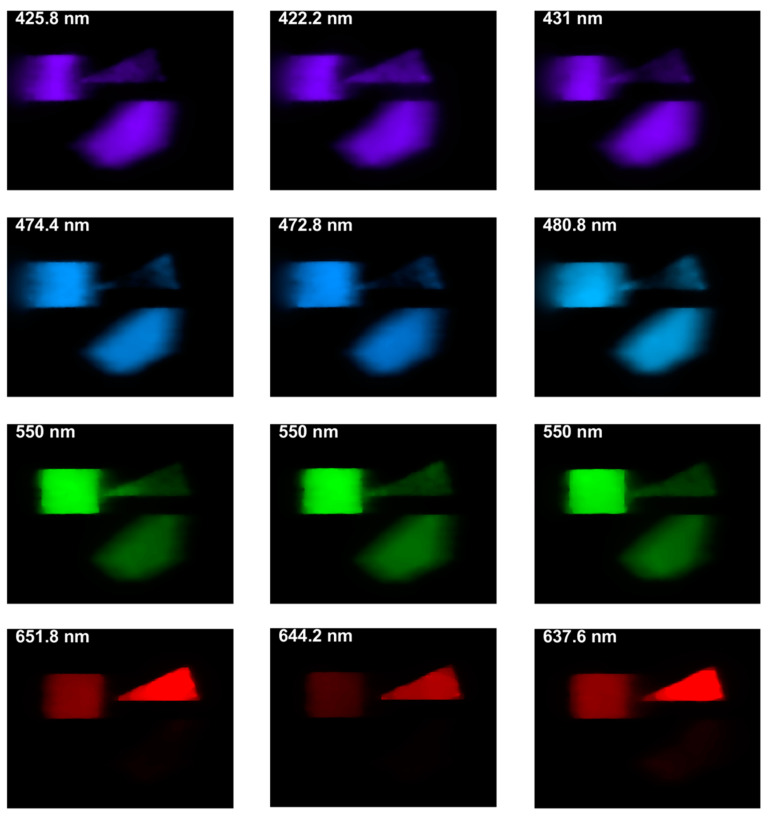
The spectral characteristic of the targets when the dual prism displacement is in Gap 1 (**first column**), Gap 2 (**middle column**) and Gap 3 (**Right column**) for four different wavelengths. Note the subtle intensity change of the colors across the rows. The images are presented using the CIE 1964 color scheme.

The presented DP-CASSI system was designed by placing the dual-prism after the relay optics to achieve an object numerical aperture of ~0.05, to cover the coded aperture dimensions of 1.664 mm and balance the Seidel aberrations. The main contribution of this work is the demonstration of wavelength tunability in the CASSI architecture by using a dual prism design. Figure 17 shows four recovered images that exhibit small changes of reflected light intensity from the targets when the air gap of the dual-prism is modulated. In all cases the shift of the spectral wavelength is with respect to the central wavelength at 550 nm. The system has been spectrally precalibrated for three different air gaps, i.e., Gap 1, Gap 2 and Gap 3 at displacements of 4.24, 4.64 and 5.04 mm, which give 51 bands, 53 bands, and 56 bands respectively. The larger number of bands (in Gap3) results from a larger degree of spectral/spatial mixing which results in a slightly larger decompression error. It can be seen from Figure 16 and Table 1 that the spectral characteristic of the red triangle object in the spectral region of 600–700 nm has been reproduced rather well when the air gaps of the DP-CASSI system are adjusted. The spectral resolution is seen to change from 5.88 nm per band for Gap 1 to 5.34 nm per band for Gap 3. Simulation for air gaps larger than 5.04 mm has not been conducted here, mainly due to significant aberrations in the system and the large number of dispersed channels for air gaps larger than 5 mm. Figure 13 highlights the effects of the regularizer tuning parameter for the decompression of the red triangle using a range of τ varying from 0.01 to 0.5. It was observed that the regularizer tuning parameter is quite sensitive to the decompression and affects the spectral accuracy rather significantly.

## 6. Conclusions

The design of a coded aperture snapshot imaging system (CASSI) capable of selectively tuning the spectral wavelength by the variation of air gap within a dual-prism is described in this paper. The motivation for this work was to study the advantages and drawbacks of tuning the wavelengths of a spectral imaging system through the modulation of the dispersive element. It was found that, while wavelength tuning in the CASSI system could be achieved by a dual prism design, correction of chromatic aberration is not straightforward. This paper reports spectral tuning of less than 1 nm in the short wavelength region (400–550 nm) and 2–12 nm in the long wavelength region (550–700 nm) through modulation of the air gap in the dual-prism from 4.24 mm to 5.04 mm. The main result of the paper is presented in Figure 17, which demonstrates the feasibility of wavelength tuning by a dual prism. Future work includes experimental validation of the design, employing reflective optics for improving dispersion linearity and aberration reductions, and considering alternative decompression algorithms to improve the robustness of the reconstruction.

## Figures and Tables

**Table 1:** For the selected groups in Figure 16, the wavelength tuning of the DP-CASSI between 600 and 680 nm in changing the air gap from Gap 1 to Gap 3.

Circular Group Index	Gap 1 4.24 mm	Gap 2 4.64 mm	Gap 3 5.04 mm	$\Delta \lambda_{gap1,\;gap2}$	$\Delta \lambda_{gap2,\;gap3}$
1	604 nm	610.4 nm	616.3 nm	6.4 nm	5.9 nm
2	614 nm	621 nm	626.7 nm	7.0 nm	5.7 nm
3	638.7 nm	644.2 nm	649.2 nm	5.5 nm	5.0 nm
4	665.8 nm	670.4 nm	674.6 nm	4.6 nm	4.2 nm
